# Efficacy and Tolerability Evaluation of a Nutraceutical Composition Containing Vitex agnus-castus Extract (EVX40™), Pyridoxine, and Magnesium in Premenstrual Syndrome: A Real-World, Interventional, Comparative Study

**DOI:** 10.7759/cureus.42832

**Published:** 2023-08-02

**Authors:** Varun P Sureja, Dharmeshkumar B Kheni, Vishal P Dubey, Jignesh Kansagra, Zeel K Soni, Sandipkumar P Bhatt, Akash Mathuria, Shrikalp S Deshpande

**Affiliations:** 1 Department of Scientific and Medical Affairs, Sundyota Numandis Probioceuticals Pvt. Ltd., Ahmedabad, IND; 2 Department of Pharmacy Practice, K.B. Institute of Pharmaceutical Education and Research, Gandhinagar, IND; 3 Department of Pharmacology, K.B. Institute of Pharmaceutical Education and Research, Gandhinagar, IND

**Keywords:** menstrual pain, vitamin b6, quality of life, ezedayz®, magnesium, pyridoxine, vitex agnus castus, premenstrual syndrome

## Abstract

Background

Pre-menstrual syndrome (PMS) is a condition associated with altered hormone levels during the menstrual phase of females and is characterised by physical, emotional, and behavioural symptoms that have a negative impact on the quality of life of females. The symptoms of PMS may vary between individuals, but the major complication is pain, especially during menstrual days. The current treatment strategy involves the use of hormonal therapies and analgesics for symptomatic relief, but these therapies have a risk of potential side effects. The use of herbal and nutraceutical supplements in PMS conditions is increasing due to their long-term safety and proven efficacy. The current real-world study aimed to evaluate the efficacy and tolerability of Ezedayz^®^ tablets containing *Vitex agnus-castus* extract (EVX40™), vitamin B6, and magnesium in PMS subjects.

Methodology

A real-world, open-label study was conducted involving 64 participants with varied severity of PMS symptoms. Participants were categorised into Group A (N=23) receiving standard therapies, Group B (N=20) receiving Ezedayz^® ^tablets, or Group C (N=21) receiving standard and Ezedayz^® ^therapy. Standard therapies were provided as per physician supervision, and Ezedayz^®^ tablets were given for 90 days. All subjects were evaluated on core symptoms of PMS like menstrual backache, menstrual cramps, joint or muscle pain, and headache using the numerical rating scale (NRS), and quality-of-life (QoL) was evaluated using a QoL questionnaire. A spontaneous reporting methodology was used to evaluate the tolerability of the therapies provided. Statistical analysis was performed as per the statistical plan. A p-value of <0.05 was considered statistically significant.

Results

Out of 64 participants, five were lost to follow-up, and the data of 59 participants were included in the final analysis. All groups showed improvement in all evaluated parameters, but Group B and Group C showed greater improvement at the end of the study in all evaluated parameters. The quality-of-life assessment revealed greater improvement in Group B and Group C participants compared to Group C in all evaluated QoL parameters. No serious side effects were observed in any subjects.

Conclusion

The results of the current study conclude that the nutraceutical composition of *Vitex agnus-castus* extract, vitamin B6, and magnesium is effective in reducing the severity of PMS symptoms and improving the quality of life of PMS subjects. The nutraceutical therapy provided greater relief from PMS symptoms compared to standard therapy alone, and this effect was augmented when the nutraceutical therapy was provided in combination with standard therapies. Similarly, the improvement in quality-of-life parameters was greater in subjects treated with nutraceuticals alone or in combination therapy. Despite the limitations of the study, the results of the current study are promising, and the nutraceutical composition (Ezedayz^®^) can be effectively used in clinical settings to control symptoms and improve the quality of life of PMS patients.

## Introduction

Premenstrual disorder (PMD) is a collective term used to indicate two identical conditions, namely premenstrual syndrome (PMS) and premenstrual dysphoric disorder (PMDD), generally characterised by somatic and psychological symptoms [1]. The overall prevalence of PMS in India ranges from 14.3% to 74.4%, while that of PMDD ranges from 3.7% to 65.7% [2]. Despite its prevalence, the exact pathophysiology of PMD is still unknown. The cyclical changes in estrogen and progesterone levels during the menstrual phase might be a possible cause of PMD symptoms [3]. Premenstrual disorder is associated with a wide range of symptoms, which are divided into two categories: affective symptoms (anger, anxiety, confusion, depression, irritability, and social withdrawal) and somatic symptoms (abdominal bloating, breast tenderness or swelling, headache, joint or muscle pain, swelling of extremities, and weight gain) [3]. As per the guidelines of the American College of Obstetrics and Gynecologists, the presence of any one affective or somatic symptom during the five days prior to menstruation from the previous three menstrual cycles is diagnosed as PMS [3]. Premenstrual disorders have a severe impact on the quality of life (QoL) of women, and their management involves various therapies like lifestyle changes, psychological therapies, and pharmacological therapies, which focus mainly on relieving the symptoms [1,3].

The current treatment options include non-pharmacological treatment (including lifestyle modifications and cognitive behaviour therapy) and pharmacological treatment. Pharmacological treatment includes oral contraceptives, psychotropic agents (like selective serotonin reuptake inhibitors and tricyclic antidepressants), and gonadotrophin-releasing hormone (GnRH) analogues. Surgical methods are also available, but they are only considered a last-resort treatment option [4]. Hormonal therapies are considered the main therapeutic option, as these therapies act by suppressing the release of luteinizing hormone (LH) and follicle-stimulating hormone (FSH), thereby modulating ovarian functions [5]. The use of painkillers and psychotropic agents is based on improving functional and mental well-being rather than working on hormonal pathways [5]. Besides these therapies, the use of dietary supplements and nutraceuticals is also considered a useful therapeutic approach for relieving PMS symptoms. Dietary supplementation includes the use of myoinositol, calcium supplements, vitamin E, magnesium, and pyridoxine supplements [5]. Herbal supplements and nutraceuticals include the use of *Vitex agnus-castus* (chaste tree extract), *Hypericum perforatum* (St. John’s wort), *Crocus sativus* (saffron), *Gingko biloba *(ginkgo), *Justicia pectoralis *(Willow herb), and evening primrose oil [5].

*Vitex agnus-castus* (VAC) is a plant native to the Mediterranean region that has been used for centuries to treat various gynaecological conditions, including PMS [6,7]. With the exact mechanism of action not fully understood, VAC is believed to work by modulating the level of hormones involved in the menstrual cycle, especially increasing the progesterone level, by acting on the hypothalamic-pituitary-gonadal axis that increases the secretion of LH, which in turn stimulates the production of progesterone [8]. Various clinical studies have previously demonstrated the effectiveness of VAC supplementation for various symptoms of PMS and PMDD, including irritability, anxiety, breast tenderness, and bloating [9]. Also, VAC is generally safe to consume with no serious adverse effects [9]. Pyridoxine, also known as vitamin B6, has been clinically evaluated for its potential role in the management of PMS symptoms. By improving serotonin and dopamine levels in the brain, pyridoxine is effective in alleviating certain emotional symptoms of PMS, including irritability, mood swings, depression, and anxiety [10,11]. As pyridoxine plays an important role in various physiological processes in the body, its supplementation is also associated with improvement in certain physical symptoms of PMS, like reduction in abdominal bloating, breast tenderness, and oedema [11]. Magnesium is a vital mineral that is essential for various physiological processes in the body. By modulating the level of various hormones (mainly progesterone) and neurotransmitters (like serotonin), magnesium supplementation provides an improvement in various PMS symptoms, including abdominal bloating, breast tenderness, mood swings, and anxiety [12,13].

Based on the scientific evidence of the potential efficacy and safety of *Vitex agnus-castus* extract, pyridoxine, and magnesium supplementation in PMS, the current study was conducted with the aim of evaluating the tolerability and efficacy of a marketed nutraceutical composition, Ezedayz® tablets containing ​​​​​​*Vitex agnus-castus extract *(EVX40™), pyridoxine, and magnesium, on the severity of symptoms and quality of life of subjects with PMS.

## Materials and methods

Participants

The inclusion criteria were: female subjects aged between 18 and 50 years; subjects referring to gynaecological centres due to PMS symptoms; the exclusion criteria were: pregnant and/or lactating women; post-menopausal women; subjects with active cancer or any other chronic disease conditions; and subjects who had undergone any surgery six months prior to study initiation. Subjects were screened and enrolled based on the inclusion and exclusion criteria.

Study intervention

The nutraceutical product Ezedayz®, containing EVX40™ 40mg, vitamin B6 1.9 mg, and elemental magnesium 250 mg per tablet, was used as the interventional product. The preferred dosing regimen was one tablet per day, either in the morning or evening, preferably before food consumption.

Study design

The study was a real-world, open-label, interventional, comparative study. The study protocol and related documents were reviewed and approved by the K.B. Institute of Pharmaceutical Education and Research Ethics Committee (protocol number: KBIEC/2021/165). After being informed of the study objectives and obtaining consent, the subjects were enrolled in the study. Initially, 64 subjects with PMS symptoms were enrolled and allocated into three groups by the study investigator. Subjects in Group A (N=23) were treated with a standard therapeutic regimen as per medical supervision; subjects in Group B (N=20) were treated with the interventional nutraceutical product; and subjects in Group C (N=21) were treated with the interventional nutraceutical product along with standard therapy. Evaluation parameters were assessed at the baseline visit and documented in a pre-designed Case Report Form (CRF). Follow-up was done on days 30, 60, and 90 after therapy allocation.

Assessment

The primary evaluation parameter evaluated the efficacy of the allocated therapy on the severity of symptoms of menstrual backache, menstrual cramps, joint and/or muscle pain, and headache using the numerical rating scale (NRS) score. The severity of symptoms is scored from 0 (no symptoms) to 10 (severe symptoms) and was evaluated at baseline (day 0), day 30, day 60, and day 90 of the study duration. Secondary evaluation parameters evaluated the efficacy of allocated therapies on improvement in quality of life (QoL) parameters using a QoL questionnaire. The QoL parameters included anxiety, mood swings or irritability, headaches, fatigue, depression, insomnia, and abdominal bloating. The QoL questionnaire evaluated the severity of symptoms between scores of 0 and three, where 0 indicated the absence of symptoms and three indicated severe symptoms and was evaluated on days 0 and 90. The tolerability of the allocated therapies was evaluated using the spontaneous reporting method.

Statistical analysis

The data were expressed as the mean ± standard deviation for all the evaluated parameters. Statistical analysis was performed using Prism software 9.0 (GraphPad Software, Boston, MA, USA). The data on individual symptom severity scores were analysed using a one-way analysis of variance (ANOVA), followed by post hoc analysis using Dunnett’s multiple comparison test. Between-group analysis was conducted using unpaired t-test analysis. A p-value of <0.05 was considered statistically significant.

## Results

Demographic data of participants

**Figure 1:**
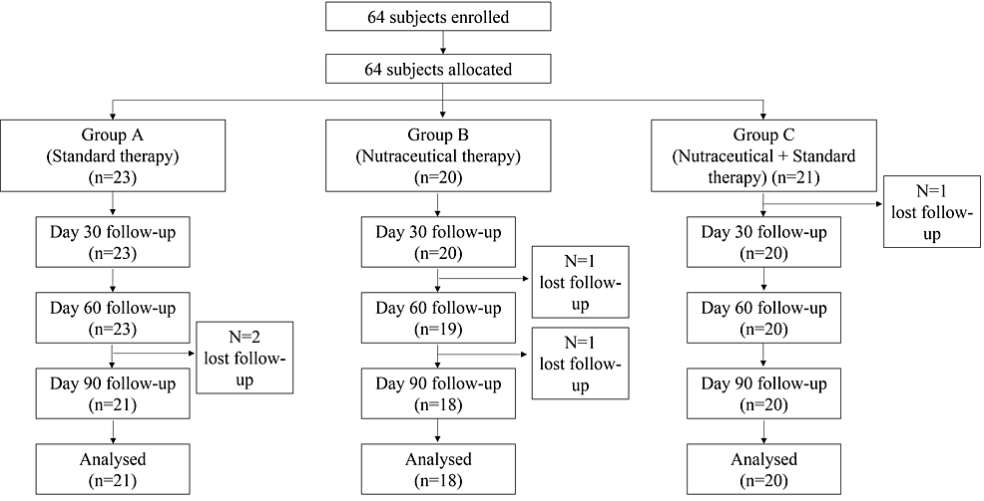
The flow of participants through the entire study duration

Initially, 64 subjects were enrolled, and a total of five subjects were lost to follow-up (two subjects from Group A, two subjects from Group B, and one subject from Group C; all subjects did not return to the study centre at the follow-up visit). The final analysis included 59 subjects, out of which 21 were from Group A (mean age: 25.52 ± 5.23 years), 18 were from Group B (mean age: 25.06 ± 4.62 years), and 20 were from Group C (mean age: 26.55 ± 5.73 years). The complete flow of participants throughout the study is presented in Figure 1.

Efficacy of interventions on primary evaluation parameters

Menstrual Backache

**Figure 2:**
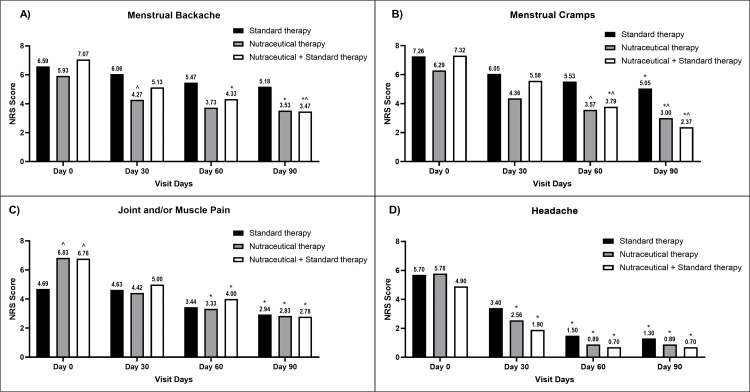
A bar diagram represents the reduction in the severity of (A) menstrual backache (B) menstrual cramps (C) joint and/or muscle pain (D) headache in the individual therapy group The data are presented as mean values. Within-group analysis was performed using one-way ANOVA with post hoc Dunnett’s multiple comparison test, and between-group analysis was performed using unpaired t-test analysis. *,^ indicates p<0.05 compared to the baseline value and p<0.05 compared to the standard therapy alone group, respectively. NRS: numerical rating scale; ANOVA: analysis of variance

Initially, 47 subjects presented the symptom of menstrual backache (17 subjects in Group A, 15 subjects in Group B, and 15 subjects in Group C). All interventions resulted in a progressive reduction in the severity of menstrual backache, but the reduction was significantly greater in Groups B and C compared to their respective baseline values. At the end of the study, the highest reduction in severity was observed in Group C, which was significant compared to Group A. The result is presented in Figure 2A.

Menstrual Cramps

Fifty-two subjects were suffering from menstrual cramps (19 subjects in Group A, 14 subjects in Group B, and 19 subjects in Group C). A progressive reduction in menstrual cramp severity was observed in all groups, while the reduction was greater in Group B and Group C as compared to Group A, with the highest reduction observed in Group C subjects. The effect of individual therapies on the symptoms of menstrual cramps is presented in Figure 2B.

Joint and/or Muscle Pain

Out of 59 subjects, 37 suffered the symptom of joint and/or muscle pain (16 subjects in Group A, 12 subjects in Group B, and nine subjects in Group C). At baseline, the severity of joint and/or muscle pain was significantly lower in Group A subjects as compared to Group B and Group C, but the reduction in severity was more pronounced in Group B and Group C as compared to Group A. At the end of the study, the severity of symptoms in all three groups was almost comparable. The result is illustrated in Figure 2C.

Headache

Twenty-nine subjects were having headaches (10 in Group A, nine subjects in Group B, and 10 subjects in Group C). The severity of headaches was progressively reduced in all groups, and the reduction was significant in all evaluated groups as compared to their baseline values, respectively. The reduction followed the same trend, with the highest severity reduction observed in Group C and the lowest in Group A, but the difference between groups at each evaluated period did not reach significance. The effect of individual therapies on headache symptoms is presented in Figure 2D.

Efficacy of the intervention on quality-of-life parameters

**Figure 3:**
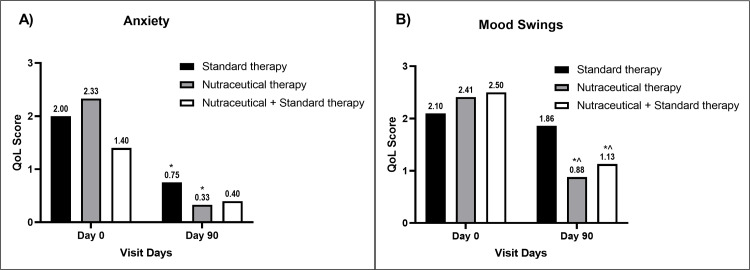
A bar diagram represents the improvement in QoL parameters of (A) anxiety and (B) mood swings in the individual therapy group The data are presented as mean values. Within-group analysis was performed using one-way ANOVA with post hoc Dunnett’s multiple comparison test, and between-group analysis was performed using unpaired t-test analysis. *,^ indicates p<0.05 compared to the baseline value and p<0.05 compared to the standard therapy alone group, respectively. QoL: quality-of-life; ANOVA: analysis of variance

The QoL parameters were progressively improved in all groups compared to their baseline values. The level of anxiety significantly decreased compared to the baseline value in Group B, while it was not significant in Groups A and C (Figure 3A).

**Figure 4:**
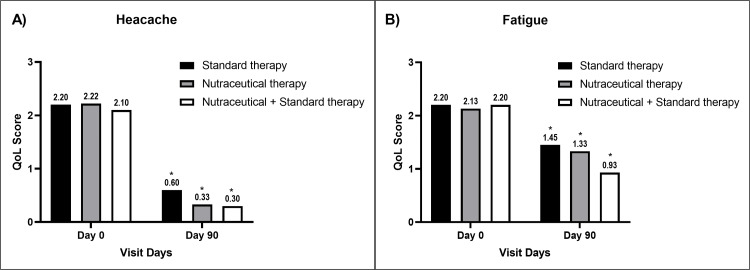
A bar diagram represents the improvement in QoL parameters of (A) headache and (B) fatigue in the individual therapy group The data are presented as mean values. Within-group analysis was performed using one-way ANOVA with post hoc Dunnett’s multiple comparison test, and between-group analysis was performed using unpaired t-test analysis. *,^ indicates p<0.05 compared to the baseline value and p<0.05 compared to the standard therapy alone group, respectively. QoL: quality-of-life; ANOVA: analysis of variance

**Figure 5:**
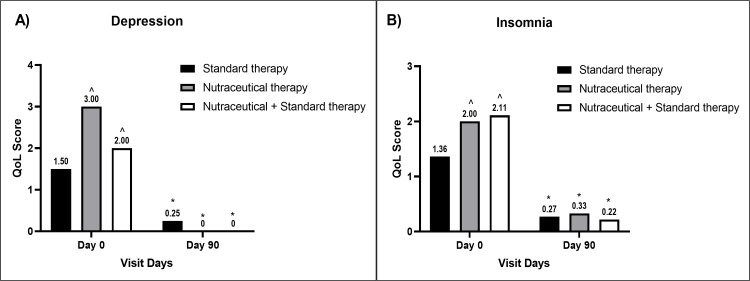
A bar diagram representation of improvement in QoL parameters of (A) depression (B) insomnia in the individual therapy group The data are presented as mean values. Within-group analysis was performed using one-way ANOVA with post hoc Dunnett’s multiple comparison test, and between-group analysis was performed using unpaired t-test analysis. *,^ indicates p<0.05 compared to the baseline value and p<0.05 compared to the standard therapy alone group, respectively. QoL: quality-of-life; ANOVA: analysis of variance

Mood swings reduced significantly in Groups B and C compared to baseline values and Group A values at the end of the study, respectively (Figure 3B). Improvement in headache (Figure 4A), fatigue (Figure 4B), and depression (Figure 5A) was significant in all groups compared to the baseline values, respectively.

**Figure 6:**
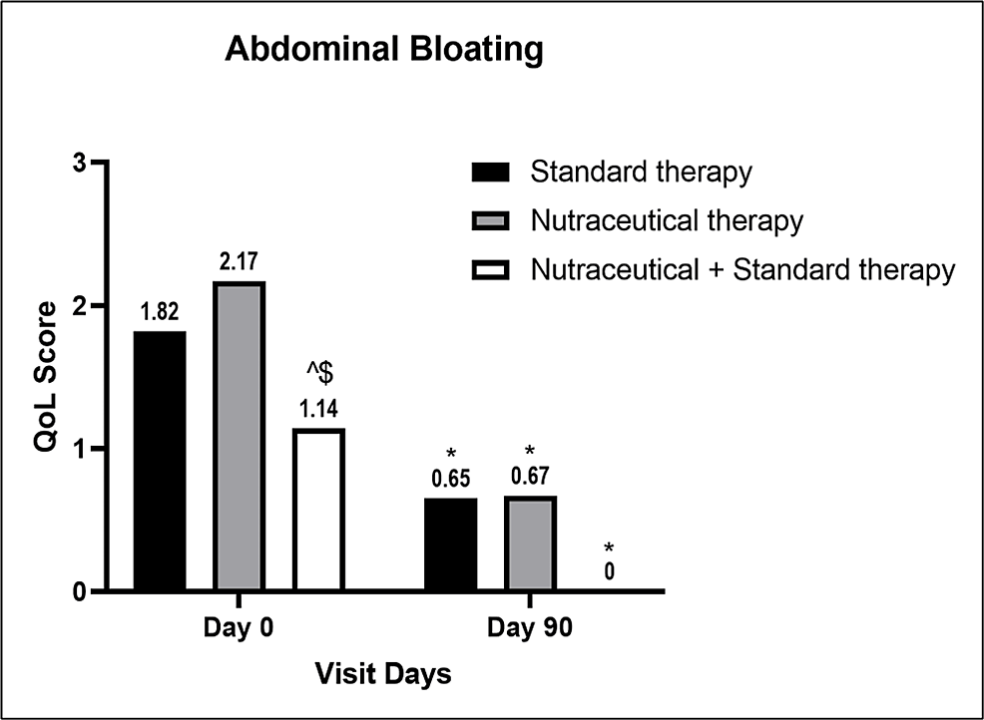
A bar diagram represents the improvement in the QoL parameter of abdominal bloating in the individual therapy group The data are presented as mean values. Within-group analysis was performed using one-way ANOVA with post hoc Dunnett’s multiple comparison test, and between-group analysis was performed using unpaired t-test analysis. *,^,$ indicates p<0.05 compared to the baseline value, p<0.05 compared to the standard therapy alone group, and p<0.05 compared to the nutraceutical therapy alone group, respectively. QoL: quality-of-life; ANOVA: analysis of variance

Improvement in insomnia was significant in Groups A and C compared to the baseline value but not in Group B (Figure 5B), while all groups had significant improvement in abdominal bloating compared to the baseline values, respectively. Group C showed significant improvement in abdominal bloating parameters compared to Group A at the end of the study (Figure 6).

Tolerability

The nutraceutical composition was well tolerated and none of the subjects in any group developed any serious side effects that could be attributed to the allocated interventions. Also, the dropouts observed during the course of the study were due to lost-to-follow-up, and none of the dropouts were due to tolerance or side-effect criteria.

## Discussion

The current study aimed to evaluate the effectiveness of a fixed-dose nutraceutical combination of *Vitex agnus-castus* extract, pyridoxine, and magnesium in reducing symptoms of PMS and improving the overall QoL of subjects with PMS. The results of the current study indicate a clear and significant benefit in terms of symptomatic reduction and improvement in the quality-of-life status of subjects with PMS.

Premenstrual syndrome (PMS) is a common condition affecting up to 80% of women of reproductive age [14]. The condition is characterised by a range of physical and emotional symptoms that typically occur a week or two leading up to a woman's menstrual period. *Vitex agnus castus*, pyridoxine (vitamin B6), and magnesium are three nutrients that have been studied for their potential role in the management of PMS. *Vitex agnus castus*, also known as chasteberry, is a plant whose extract is believed to work by modulating levels of hormones involved in the menstrual cycle, particularly by increasing levels of progesterone [8]. Pyridoxine is believed to work by increasing the level of the neurotransmitter serotonin in the brain, which can help alleviate some of the emotional symptoms of PMS [10,11]. Magnesium is believed to work by regulating levels of various hormones and neurotransmitters involved in the menstrual cycle, including estrogen, progesterone, and serotonin [12,13].

*Vitex agnus-castus* has been previously evaluated in various clinical trials for various gynaecological conditions, including PMS and PMDD. A recent systematic review and meta-analysis study evaluated the effectiveness of VAC in premenstrual syndrome. The study included 17 clinical studies, out of which data from 14 clinical studies were used for pooled analysis. The results of the study demonstrate that VAC supplementation was associated with significant improvement in PMS conditions, but the results of the study were hindered due to a high risk of bias, significant publication bias, and high heterogeneity among the included studies [15]. Another systematic review was conducted, which included the results of eight clinical studies that evaluated the effect of VAC supplementation on PMS and PMDD conditions. The result of that study indicated that VAC supplementation was effective and generally well tolerated in PMS and PMDD conditions [9].

The effect of pyridoxine supplementation on PMS symptoms has been evaluated using various clinical studies. A meta-analysis study conducted to evaluate the effectiveness of pyridoxine supplementation on PMS symptoms concluded that pyridoxine supplementation was associated with a significant reduction in the total PMS score as well as individual physical and psychological symptoms scores as compared to the control group [10]. Magnesium is an important element and is involved in various physiological processes in the body. A clinical study was conducted involving PMS subjects who were treated with magnesium supplements, pyridoxine, or placebo. The study showed that magnesium supplements were significantly effective in reducing various symptoms of PMS, like cravings, depression, water retention, anxiety, and somatic changes. The study also showed that magnesium was equally effective as pyridoxine in reducing PMS symptoms and, hence, can be considered an effective therapy for subjects with PMS [16].

The results of the current study are in line with the results of previous studies. The nutraceutical composition therapy was associated with a significant reduction in various symptoms of PMS and also improved the quality of life of the subjects. The current study has various strengths. First, it is the first study to evaluate the tolerability and effectiveness of a nutraceutical combination of *Vitex agnus-castus* extract, vitamin B6, and magnesium in PMS subjects. Secondly, the study was designed to directly compare the efficacy of nutraceutical therapy with the efficacy of standard therapeutic regimens currently used in PMS. Thirdly, this study also provides evidence of the synergistic efficacy of nutraceutical therapy when given along with a standard therapeutic regimen while having no drug interactions that might cause any serious side effects in subjects with PMS.

The current study has certain limitations, which include the open-label design of the study. Also, the study evaluated the effect of interventions for a short period of time, and hence further clinical studies with a longer duration are warranted. Lastly, the study primarily focused on the symptoms of PMS and did not evaluate the effect of nutraceutical therapy on hormonal and neurotransmitter levels, which needs to be addressed by conducting further clinical studies.

## Conclusions

The results of the current study conclude that the nutraceutical composition of Vitex agnus-castus extract, vitamin B6, and magnesium is effective in reducing the severity of PMS symptoms and improving the quality of life of PMS subjects. The nutraceutical therapy provided greater relief from PMS symptoms compared to standard therapy alone, and this effect was augmented when the nutraceutical therapy was provided in combination with standard therapies. Similarly, the improvement in quality-of-life parameters was greater in subjects treated with nutraceuticals alone or in combination therapy. Despite the limitations of the study, the results of the current study are promising, and the nutraceutical composition (Ezedayz®) can be effectively used in clinical settings to control symptoms and improve the quality of life of PMS patients.
